# Stimuli-Specific Senescence of Primary Human Lung Fibroblasts Modulates Alveolar Stem Cell Function

**DOI:** 10.3390/cells13131129

**Published:** 2024-06-29

**Authors:** Maria Camila Melo-Narváez, Nora Bramey, Fenja See, Katharina Heinzelmann, Beatriz Ballester, Carina Steinchen, Eshita Jain, Kathrin Federl, Qianjiang Hu, Deepesh Dhakad, Jürgen Behr, Oliver Eickelberg, Ali Önder Yildirim, Melanie Königshoff, Mareike Lehmann

**Affiliations:** 1 Institute of Lung Health and Immunity (LHI), Helmholtz Munich, Comprehensive Pneumology Center (CPC-M), German Center for Lung Research (DZL), 81377 Munich, Germany; maria.melonarvaez@helmholtz-munich.de (M.C.M.-N.); fenja.see@helmholtz-munich.de (F.S.); carina.steinchen@helmholtz-munich.de (C.S.); eshita.jain@helmholtz-munich.de (E.J.); deepesh.dhakad@helmholtz-munich.de (D.D.); alioender.yildirim@helmholtz-munich.de (A.Ö.Y.); 2 Institute for Lung Research, Philipps-University Marburg, German Center for Lung Research (DZL), 35043 Marburg, Germany; 3 Faculty of Health Sciences, Universidad Cardenal Herrera—CEU, CEU Universities, 46115 Valencia, Spain; 4 Division of Pulmonary, Allergy & Critical Care, and Sleep Medicine, University of Pittsburgh Medical Center, Pittsburgh, PA 15213, USA; huqj@pitt.edu (Q.H.); eickelbergo@upmc.edu (O.E.); koenigshoffm@upmc.edu (M.K.); 5 Department of Medicine V, University Hospital Munich, Medical Faculty of the LMU Munich, 81377 Munich, Germany; juergen.behr@med.uni-muenchen.de; 6 Institute of Experimental Pneumology, University Hospital Munich, Ludwig-Maximilians University, 81377 Munich, Germany; 7 Lung Aging and Regeneration, Institute for Lung Health (ILH), 35392 Giessen, Germany

**Keywords:** cellular senescence, fibroblasts, chronic lung diseases, aging

## Abstract

Aging is the main risk factor for chronic lung diseases (CLDs) including idiopathic pulmonary fibrosis (IPF) and chronic obstructive pulmonary disease (COPD). Accordingly, hallmarks of aging like cellular senescence are increased in these patients in different lung cell types including fibroblasts. However, little is known about the different triggers that induce a senescence phenotype in different disease backgrounds and its role in CLD pathogenesis. Therefore, we characterized senescence in primary human lung fibroblasts (phLF) from control, IPF, or COPD patients at baseline and after exposure to disease-relevant insults (H_2_O_2_, bleomycin, TGF-β1) and studied their capacity to support progenitor cell potential in a lung organoid model. Bulk-RNA sequencing revealed that phLF from IPF and COPD activate different transcriptional programs but share a similar senescence phenotype at baseline. Moreover, H_2_O_2_ and bleomycin but not TGF-β1 induced senescence in phLF from different disease origins. Exposure to different triggers resulted in distinct senescence programs in phLF characterized by different SASP profiles. Finally, co-culture with bleomycin- and H_2_O_2_-treated phLF reduced the progenitor cell potential of alveolar epithelial progenitor cells. In conclusion, phLF from COPD and IPF share a conserved senescence response that varies depending on the insult and impairs alveolar epithelial progenitor capacity ex vivo.

## 1. Introduction

Chronic respiratory diseases are the third leading cause of death globally [1]. Those include chronic obstructive pulmonary disease (COPD) and interstitial lung diseases such as idiopathic pulmonary fibrosis (IPF) [1]. COPD is an inflammatory disease [2] characterized by small airway remodeling, emphysema, and chronic bronchitis [3]. The main risk factors are cigarette smoking and age but exposure to air pollution or pathogens also contributes to COPD [3]. IPF is a progressive fibrosing disease characterized by excessive matrix deposition [4,5]. Higher age and exposure to cigarette smoke are the main risk factors for IPF [4,5]. Familial cases of IPF have mostly been linked to mutations in genes encoding surfactant protein C and A2 (SFTPC, SFTPA2) and telomerases (TERT and TERC), which lead to telomere shortening, cellular senescence, and exhaustion of lung stem cells [4,5,6,7]. The incidence rates for both COPD and IPF increase in the elderly population [8] and several cellular hallmarks of aging such as cellular senescence are increased in the lung tissue of both COPD and IPF patients [9,10,11,12]. Senescent cells are characterized by an irreversible cell cycle arrest (upregulation of cyclin-dependent kinase inhibitor 1A (CDKN1A/P21) and/or 2A (CDKN2A/P16), resistance to apoptosis, altered metabolism and the secretion of growth factors and pro-inflammatory cytokines known as the senescence-associated secretory phenotype (SASP) [13] that promotes inflammation and tissue remodeling.

Fibroblasts are effector cells in both diseases, causing impaired tissue structure by aberrant deposition of extracellular matrix (ECM) on the one hand, and demonstrating hallmarks of senescence on the other hand [10,11,14,15,16]. Recent progress in single-cell omics revealed the existence of disease-specific cellular subtypes in the mesenchymal compartment [17,18,19,20,21]. Although accumulation of senescent cells is observed in both COPD and IPF, it remains unclear how senescence is induced in specific cell types and whether certain subtypes are more prone to different senescence stimuli. Several studies have shown that an altered epithelial-to-mesenchymal crosstalk can lead to the structural and functional changes observed in IPF and COPD lungs [22]; however, little is known about the role of senescent fibroblasts in these processes. Therefore, here, we used transcriptomic analysis to characterize senescence programs in the mesenchymal compartment in lungs from COPD, IPF, and control lungs. Then, we used well-known senescence inducers to study the susceptibility and senescence responses of primary human lung fibroblasts (phLF) derived from these three disease backgrounds. Finally, we used an organoid assay to evaluate the effect of epithelial-to-mesenchymal crosstalk on progenitor cell capacity in vitro. In conclusion, we show that senescent fibroblasts accumulate in IPF and COPD lungs, however, after isolation and culture, phLF from different disease origins displayed a low baseline senescence. Moreover, stimuli and not disease origin determine the senescence phenotype after exposure to disease-relevant insults in phLF in vitro. Finally, senescent fibroblasts modulate the progenitor cell capacity of alveolar epithelial progenitors.

## 2. Materials and Methods

### 2.1. Ethic Statement

The study was approved by the local ethics committee of the Ludwig-Maximilians University of Munich, Germany (Ethic vote 19-630). Written informed consent was obtained for all study participants.

### 2.2. Cell Culture

Primary human lung fibroblasts (phLF) from age-matched control, COPD, and IPF patients (Table 1) were obtained from the CPC-M bioArchive at the Comprehensive Pneumology Center (CPC Munich, Germany)**.** The isolation of primary human lung fibroblasts (phLF) from lung tissue was carried out as follows: Human lung specimens were cut into 1–2 cm^2^ pieces and digested with 1 mg/mL of Collagenase I (Biochrom, Cambridge, UK) at 37 °C for 2 h. The samples were then filtered through 70 µm nylon filters (BD Falcon, Franklin Lakes, NJ, USA). Single-cell suspensions were centrifuged at 400× *g*, 4 °C for 5 min and the supernatant was discarded. The resulting pellets were resuspended in DMEM/F12 medium (Life Technologies, Carlsbad, CA, USA) supplemented with 20% fetal bovine serum (Pan Biotech, Aidenbach, Germany) and 1% penicillin/streptomycin (10,000 U/mL, Life Technologies, Carlsbad, CA, USA) and plated on 10 cm cell culture dishes. The medium was changed after 2 days and cells were split upon reaching 80–90% confluence. For this study, phLF were used in passages 4–9. Fibroblast purity was determined by CD45-/CD31-negative and CD90-positive expression between passages 1 and 7 using FACS and quantitative PCR. A pure population (negative for CD45 and CD31) was observed from passage 2 onwards). phLF were cultivated in DMEM/F-12 (Life Technologies, Carlsbad, CA, USA) with 1% penicillin/streptomycin (10,000 U/mL, Life Technologies, Carlsbad, CA, USA), and 10% Fetal Bovine Serum (PAN Biotech, Aidenbach, Germany) and medium was changed every second day. For qPCR and ELISA experiments, phLF were seeded on 6-well plates at a density of 4.5 × 10^4^ cells per well. After 24 h of incubation, treatment solutions were applied and changed every 48 h. After day 3 and day 7 of treatment, supernatants of cells were collected, centrifuged, and frozen at −80 °C. For RNA isolation, the treatment solution was removed, the wells were washed twice with DPBS, and cells were frozen directly at −80 °C. Viability and metabolic activity were determined by lactate dehydrogenase assay according to the manufacturer’s instructions (Enzo Life Sciences, Farmingdale, NY, USA). Briefly, 100 µL of supernatant from phLF-treated vehicle control, TGF-β1, H_2_O_2_, or bleomycin were mixed 1:1 with the working solution. After 30 min of incubation at room temperature, stop solution was added and absorbance was measured at 490 nm using a microplate reader Sunrise (Tecan GmbH, Männedorf, Switzerland).

### 2.3. Induction of Cellular Senescence

PhLF (passages 3–9) were exposed to 5 ng/mL recombinant human TGF-β1 (R&D Systems, 240-B-002), 180μM H_2_O_2_ (Sigma-Aldrich, Darmstadt, Germany) or 3.3 mU/mL bleomycin sulfate (Sigma-Aldrich, Darmstadt, Germany) in culture medium with 5% FBS and treatment was replenished every 48 h. Negative control solutions contained an equivalent volume of 0.1% BSA in PBS (TGF-β1), plain medium (H_2_O_2_), or DPBS (bleomycin).

### 2.4. Organoid Assay

Murine distal lung epithelial cells (CD45−-/CD31−-/EpCAM+) were obtained after enzymatic and mechanical digestion of mouse lungs using a medium containing dispase for 45 min [23]. Single-cell suspension was sequentially filtered using 100 µm and 40 µm nylon filters. Cells were then sorted via magnetic-assisted cell sorting (MACS), sorting first to deplete CD45 and CD31 positive cells. Subsequently, the flow-through was MACS sorted to enrich for EpCAM+ cells. Meanwhile, phLF were treated as previously described and after 7 days, control and senescent phLF were treated with Mitomycin C (10 µg/mL) for 2 h, at 37 °C and 5% CO_2_ to stop proliferation. Then, phLF were washed with 1X DPBS and kept in fresh medium for at least 1 h at 37 °C and 5% CO_2_. phLF and murine distal lung epithelial cells were mixed in a 1:1 ratio (10,000 cells each) in Matrigel (Corning, Somerville, MA, USA) and 50 µL of the mix was added per well in 96-well plates as previously described [23]. Plates were incubated for 10–15 min at 37 °C and then organoid medium supplemented with Rock inhibitor (Ri) was added for the first 48 h. After this, organoid medium without rock inhibitor was used for medium change every 2–3 days [23]. After 14 days, organoids were imaged using a LifeCellImager Observer Z1 microscope (Zeiss, Oberkochen, Germany). Maximum projections were generated on Zen Blue software v.3.0 (Zeiss, Oberkochen, Germany). Organoid size and number were determined using the Napari organoid counter v.0.4.18 [24,25]. Data and plots were generated and analyzed in GraphPad Prism 9.5.1.

### 2.5. RT-qPCR

RNA was isolated using the peqGOLD Total RNA Kit (VWR International, Radnor, PA, USA) according to the manufacturer’s instructions. For cDNA synthesis, mastermix was prepared by mixing: Random Hexamers (10 uM, Invitrogen, Waltham, Massachusetts, USA, cat.# 100026484), dNTP Mix (2 mM, Invitrogen, Waltham, MA, USA, cat.# R0192), 5× First-strand buffer (1X, Invitrogen, Waltham, MA, USA, cat.# Y02321), 0.1 M DTT (40 mM, Invitrogen, Waltham, MA, USA, cat.# Y00147), RNAse Inhibitor (20 U/uL, Applied Biosystems, Waltham, MA, USA, cat.# N8080119), and M-MLV Reverse Transcriptase (10 U/uL, Invitrogen, Waltham, MA, USA, cat.# 28025013) using the following reverse transcription program: 1 cycle at 20 °C for 10 min, 1 cycle at 43 °C for 75 min, and 1 cycle at 99 °C for 5 min. RT-qPCR mix was prepared using 1X Light Cycler 480 SYBR Green Master (Roche, Basel, Switzerland) and primer pairs at 5 µM (Table 2). Then, samples were run in a Light Cycler 480II (Roche, Basel, Switzerland): 1 cycle at 50 °C for 2 min, 1 cycle 95 °C for 5 min, followed by 45 cycles of 1X 95 °C for 5 s, 1X 59 °C for 5 s, 1 × 72 °C for 5 s. The delta Ct values were determined by a two-derivative method and log2FoldChange were calculated based on the 2–∆∆C method [26].

### 2.6. ELISA

ELISA for IL-6 (DY206), GDF-15 (DY957), Serpin E1/PAI-1 (DY1786), and total MMP-3 (DY513) were performed according to manufacturer’s instructions (R&D Systems Minneapolis, Minnesota, USA). Absorbance was measured at 450 nm using the microplate reader Sunrise (Tecan GmbH, Männedorf, Switzerland). The final absorbance values were calculated by subtracting the background signal. The concentrations were calculated using interpolation of a linear regression based on the standard curve. Concentrations were normalized to the total protein content of the cell lysate at the final collection time point. Heatmaps display the average of fold changes (treatment/control) of at least 3 biological replicates.

### 2.7. Senescence Associated β-Galactosidase Staining

phLF were seeded (8.0 × 10^3^ cells/well) and the Senescence β-galactosidase staining Kit from Cell Signaling (9860) was used according to the manufacturer’s instructions to determine senescence induction after day 3 and day 7 of treatment. Briefly, on collection days, cells were washed once with 1× PBS and fixed with 1× fixative solution for 15 min at RT. Cells were then rinsed twice with 1× PBS and incubated in β-Galactosidase Staining Solution (pH of 6.0) overnight at 37 °C in a dry incubator (no CO_2_). The next day, cells were washed twice with 1× PBS and kept on 70% glycerol at 4 °C until imaging. A bright-field microscope was used to obtain at least 3 regions of interest at 100× magnification per sample. Then, the number of positive cells and total cell number were counted manually using ImageJ v.1.54. Fold change was calculated as treated/untreated.

### 2.8. Immunofluorescence Staining

Organoids were fixed with ice-cold methanol and 2D cell cultured cells were fixed with 4% PFA. Then, samples were blocked with 5% donkey normal serum in 0.1% PBST for 1 h at RT and then incubated with primary antibodies (Table 3) diluted in 1% donkey normal serum at 4 °C overnight. Samples were washed 3× for 20 min (organoids) or 5 min (cells) with 0.1% PBST and then incubated for 2 h at RT with secondary antibodies (Table 3) plus DAPI. Cells were then washed again 3 times and mounted with a fluorescence mounting medium (Dako, Agilent, Santa Clara, California, USA). The mean fluorescence intensity of images taken with an LSM 710 Confocal microscope (Zeiss, Oberkochen, Germany) was quantified using ImageJ v.1.54. Percentages of positive cells were calculated using QuPath v0.4.3 [27].

### 2.9. Bulk-RNA Sequencing

Primary human lung fibroblasts were isolated from the control donor, IPF, and COPD lungs, cultured until passage 2 as previously described [28,29], and used for total RNA isolation. Bulk-RNA sequencing was performed by IMGM Laboratories GmbH. For this, the Illumina TruSeq^®^ Stranded Total RNA (including Ribo-Zero) Gold technology was used to generate rRNA-depleted total RNA libraries from RNA samples with good integrity and concentration (Nanodrop and Bioanalyzer). Sequencing was performed on the Illumina NextSeq^®^500 next-generation sequencing system using 2 × 150 bp paired-end read chemistry. Adapter trimming and filtering of low-quality reads were performed using Cutadapt (v2.10). HISAT2 was used to align reads against the GRCh38 reference genome. Mapped reads were quantified using HTseq (v0.13.5) with the setting “–stranded reverse”. The gene annotation used for quantification was Ensembl version 108. Quality controls and downstream analysis were performed in R v.4.3.2. Raw counts were corrected for batch bias due to biological variance using the ComBatseq function and due to unknown variables calculating surrogate variables on the surrogate variable analysis (sva) package v.3.50.0 [30]. Then, differential expression analysis was performed using the DESeq2 package v.1.42.0 (pAdjustMethod = ”BH”, alpha = 0.05) and shrinkage of the log-fold change (LFC) [30]. Differentially expressed genes (adjusted *p*-value < 0.05, LFC > 0) were extracted and used for downstream analysis and data exploration [30]. Raw counts were normalized according to library size using the DeSeq2 package v.1.42.0 and global Z-scores were calculated before hierarchical clustering to generate heatmaps.

### 2.10. Analysis of Human Lung COPD Single Cell RNA Sequencing Data

Mesenchymal cells from both donor and COPD patients were obtained from a single-cell RNA sequencing dataset available under Gene Expression Omnibus (GEO) accession number “GSE136831”. The original annotations from the authors [17] were used for the analysis. The data was processed using the Seurat Package in R (4.3.1) and a Seurat v3 Assay Object (options(Seurat.object.assay.version=“v3”)) was created using the CreateSeuratObject function with min.cells set to 5 and min.features set to 500. A total of 165,703 cells were obtained after filtering, of which 2078 mesenchymal cells were analyzed. The data was dimensionally reduced using Uniform Manifold Approximation and Projection (UMAP) with the top 30 principal components using the RunUMAP function with a seed value set to 43 (set.seed = 43) and represented using the DimPlot function. Dotplots and Featureplots for the genes of interest were plotted using the scCustomize (v2.0.1) package in R.

### 2.11. Analysis of Human Lung IPF Single Cell RNA Sequencing Data

Mesenchymal cells from both donors and IPF patients were extracted from a single-cell RNA sequencing dataset available under Gene Expression Omnibus (GEO) accession number “GSE135893”. The annotations from Mayer, C. et al. [31] were used for the analysis. The data was processed using Scanpy version 1.9.3. A total of 94,839 cells of donors and IPF were obtained after filtering, of which 2278 mesenchymal cells were analyzed. The data was dimensionally reduced using Uniform Manifold Approximation and Projection (UMAP) with the top 50 principal components using the function of scanpy.pp.neighbors() and scanpy.tl.umap(). The cell type and gene expression profiled were shown using the function of scanpy.pl.umap().

### 2.12. Microarray Analysis

To characterize the gene expression of senescence-associated genes, we analyzed a publicly available microarray dataset from whole lung homogenate from subjects undergoing thoracic surgery, which have been previously classified as controls, having interstitial lung disease, or COPD by clinical history, CT scan, and pathology. For the analysis, only patients diagnosed with UIP/IPF or COPD (Gold Stage II-IV) were included. Microarray log2 transformed and normalized expression was used for differential expression analysis with Limma package in R v.3.58.1 (*p*-value adjusted by Benjamini & Hochberg Method) and differentially expressed genes were plotted in a Venn Diagram in R v.4.3.2 [30]. Normalized expression values and predicted diffusing capacity of the lungs for Carbon Monoxide (dclo, %) were used for correlation analysis in GraphPad Prism 10.2.3.

### 2.13. Data Collection and Analysis

Primary human fibroblasts from Donor, COPD, and IPF were used for different experiments in this study (Table 1). For titration and organoid assays phLF only from control donors were used and single points represent different biological or technical replicates as indicated in figure legends. To analyze the capacity of the triggers used to induce senescence we pooled together the data collected using all the different samples listed in Table 1. Here, single points represent biological replicates and points shape indicate the disease origin. Finally, to study differences linked to the background disease we separated the samples listed in Table 1 into three different groups and used the data collected with these same samples for downstream analysis. Here, single points represent biological replicates and points shape indicate the disease origin.

## 3. Results

### 3.1. Senescent Fibroblasts Accumulate in IPF and COPD Lungs

**Figure 1:**
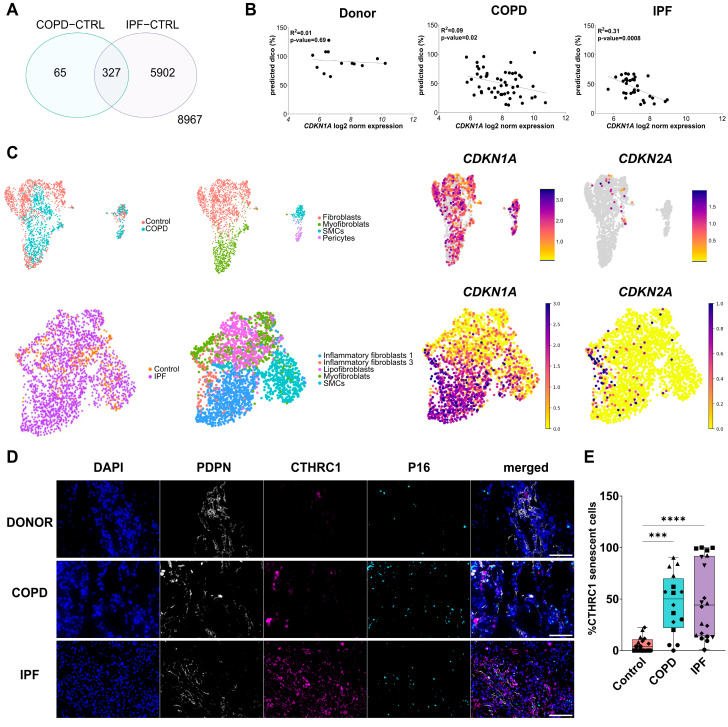
Senescent cells are enriched in IPF and COPD lungs. (**A**) Microarray analysis revealed several differentially expressed genes in common between COPD and IPF, when compared to healthy donor controls. (**B**) Pearson correlation analysis of predicted DLCO (%) and *CDKN1A/P21* expression in Donor, IPF, and COPD lungs. Single points represent single patients. *p*-value < 0.05. (**C**) UMAP representation of the mesenchymal compartment and corresponding *CDKN1A* and *CDKN2A* expression in Control, COPD, and IPF lungs. (**D**) Representative images of immunofluorescence staining for Podoplanin (PDPN), CTHRC1, and CDKN2A/P16 in control, IPF, and COPD lungs. Scalebar = 100 µm. (**E**) Quantification of CTHRC1+ P16+ double positive cells in control, IPF, and COPD lungs. Single points with similar shapes represent single images from 4 to 5 different biological replicates. *** *p*-value < 0.001, **** *p*-value < 0.0001. Kruskal-Wallis test, followed by Dunn´s multiple comparisons test.

To analyze senescence in IPF and COPD, we first explored a publicly available dataset from COPD, IPF, and control lungs. We found that despite only a subset of DEGs overlapping in COPD and IPF (Figure 1A), the gene expression of the senescence marker *CDKN1A/P21* is negatively correlated with predicted DLCO in age-matched COPD and IPF patients, suggesting that the degree of senescence correlates with disease severity in both diseases (Figure 1B). To investigate whether this is mediated in part by the mesenchymal compartment, we took advantage of publicly available single-cell RNA sequencing datasets. Here, we identified cellular senescence as marked by *CDKN1A/P21* and *CDKN2A/P16* expression in specific fibroblast subtypes in COPD and IPF patients (Figure 1C). To validate the clinical relevance of these findings, we stained lung tissue sections derived from Control, IPF, and COPD patients for Podoplanin (PDPN), a structural marker for alveolar epithelium and collagen triple helix repeat containing 1 (CTHRC1), a recently described marker for fibroblasts [32] in combination with the senescence marker CDKN2A/P16. Here, we found a significant increase in CTHRC1+ CDKN2A/P16 + double positive cells in COPD and IPF lungs, when compared to age-matched healthy controls (Figure 1D,E).

### 3.2. Senescence Program in phLF Derived from Different Diseases Backgrounds

**Figure 2:**
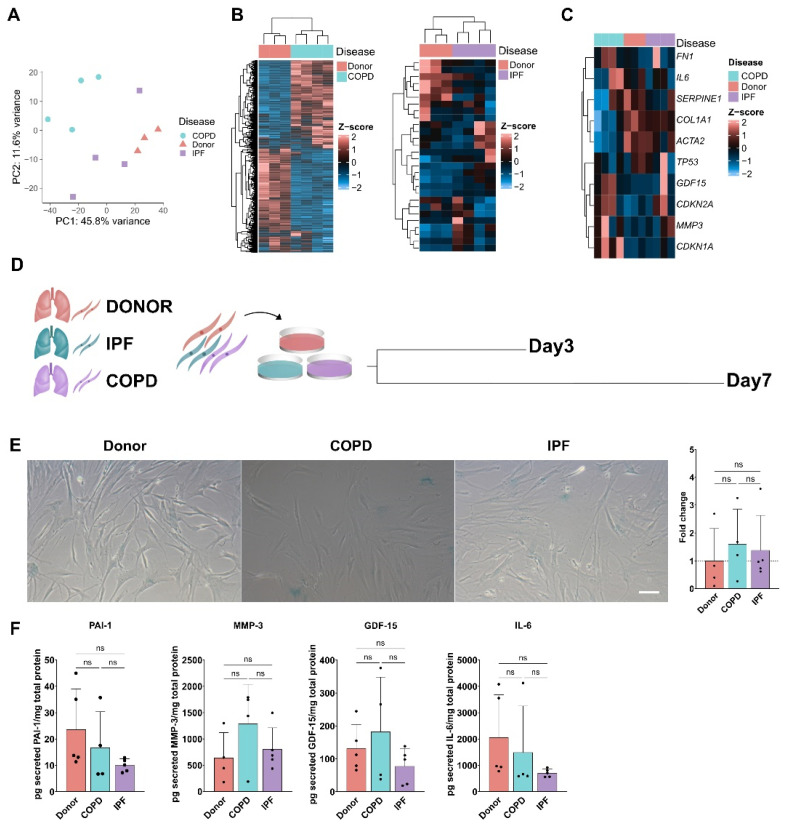
Primary human fibroblasts from different disease backgrounds share a similar senescence phenotype at baseline. (**A**) Principal component analysis after bulk-RNA sequencing showed distinct phenotypes of phLF isolated from IPF, COPD, or control lungs. Single points represent different biological replicates. (**B**) Hierarchical clustering of differentially expressed genes of phLF isolated from IPF, COPD, or control lungs. (**C**) Hierarchical clustering of normalized counts for senescence- and fibrosis-associated genes expressed by phLF isolated from IPF, COPD, or control lungs. (**D**) Experimental design to characterize senescence in primary human fibroblasts from Donor, COPD, and IPF patients after short (Day 3, d3) and prolonged (Day 7, d7) culture. (**E**) Representative images and quantification of SA-β-galactosidase staining of phLF from Donor, COPD, and IPF. Data points represent an average of 3 different regions of interest of at least 3 different biological replicates. (**F**) ELISA for SASP factors in supernatants from phLF that were cultured for 7 days. Data points represent different biological replicates of the concentration of each secreted protein (pg/mL) normalized to total cell protein content (mg/mL). All *p*-values (<0.05) were calculated based on the Kruskal–Wallis test, followed by Dunn´s multiple comparisons test.

Since we observed an accumulation of senescent fibroblasts in the lung from COPD and IPF patients, we next wanted to investigate the specific senescence programs in phLF isolated from controls, COPD, or IPF lungs. Bulk-RNA sequencing revealed that phLF from COPD, IPF, and Control lungs have a distinct phenotype as shown by the principal component analysis (Figure 2A). Notably, IPF-derived phLF showed less transcriptomic variation with only 50 differentially expressed genes (DEG) than COPD-derived phLF (1647 DEG) compared to control lungs (Figure 2B). Nevertheless, IPF- and COPD-derived phLF showed increased expression of the well-known senescence-related markers: *CDKN1A/P21*, *CDKN2A/P16*, tumor suppressor protein 53 (*TP53*), Growth Differentiation Factor 15 (*GDF-15*), and Matrix Metalloproteinase 3 (*MMP3*) (Figure 2D), although not reaching statistical significance. Moreover, COPD-derived phLF displayed lower levels of Epithelial-to-mesenchymal (EMT) markers such as smooth muscle alpha (α)-2 actin (*ACTA2*) and Collagen 1 (*COL1A1*) as shown in Figure 2C. To better understand the senescence programs in the different diseases and how they change over culturing time, we characterized multiple well-accepted senescence markers in phLF from COPD, IPF, and Control lungs after short (Day 3, Figure S1) and prolonged culture (Day 7, Figure 2D).

Here, we found a similar percentage of SA-β-galactosidase (SA-β-gal)+ cells in all three groups after 7 days of culture (Figure 2E). Moreover, phLF secreted several SASP components (Plasminogen Activator Inhibitor 1 (PAI-1), MMP-3, GDF-15, and Interleukin 6 (IL-6)) indistinctively from the disease background after 7 days of culture (Figure 2F). This together suggests that phLF derived from COPD, IPF, and control lungs share a similar senescence program in vitro, contrary to the enriched senescence observed in situ in COPD and IPF lungs when compared to control lungs.

### 3.3. Disease Relevant Stimuli Can Induce Senescence in phLF

**Figure 3:**
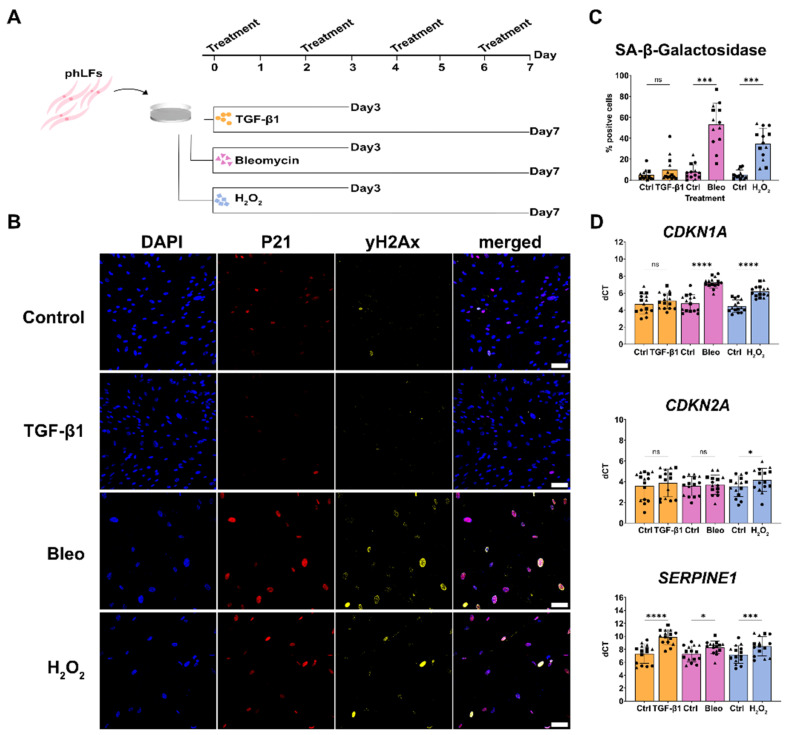
Induction of senescence in primary human fibroblasts with disease-relevant stimuli. (**A**) Experimental design to characterize senescence induction on phLF from Donor, COPD, or IPF patients after treatment with TGF-β1, H_2_O_2_, or bleomycin, respectively. (**B**) Representative images of immunofluorescence staining for senescence-related markers (CDKN1A/P21) and yH2Ax on phLF after 7 days of treatment with TGF-β1, H_2_O_2_, or bleomycin. (**C**) Quantification of SA-β-galactosidase activity after 7 days of treatment. (**D**) qRT-PCR to assess gene expression of senescence-related markers after treatment with H_2_O_2_, bleomycin, and TGF-β1. Data points represent biological replicates from donor (square), IPF (circle), and COPD (triangle). * *p*-value < 0.05: Friedman paired test followed by Dunn´s multiple comparisons test. * *p*-value < 0.05, *** *p*-value < 0.01, **** *p*-value < 0.001. Friedman paired test followed by Dunn´s multiple comparisons test.

Given the low baseline senescence in phLF isolated from COPD and IPF lungs, we next aimed to induce senescence using disease-relevant triggers. Aging and exposure to cigarette smoke are the main risk factors for IPF and COPD and have been linked to stress-induced senescence [22]. Therefore, we exposed phLF to hydrogen peroxide (H_2_O_2_) or bleomycin, since they induce the release of reactive oxygen species (ROS) as well as genomic DNA damage as observed in the lungs of smokers [33,34]. Moreover, we included transforming growth factor beta 1 (TGF-β1), a well-known profibrotic mediator, as the third stimulus, given that previous studies showed that TGF-β1 not only promotes fibroblast activation but also senescence in vitro [33]. For bleomycin and H_2_O_2_, we tested several doses and observed a dose-dependent induction of SA-β-gal activity (Figure S2A,B). A dose of 3.3 mU/mL for bleomycin and 180 µM for H_2_O_2_ was used for further experiments, since these doses induced a high percentage of senescent cells (58.8% and 61.5%, respectively, Figure S2) with a significant reduction in cell proliferation (Figures S2 and S3) and without a sustained effect on cell death (Figure S6), well-known characteristics of senescent cells. Next, we tested whether single (S) or repetitive (R) treatment would induce different responses in senescence-related markers. Here, we did not find any significant difference between both treatment regimes, but the repetitive treatment resulted in higher induction of *CDKN1A/P21* (Figure S2D), and therefore, we continued with this treatment scheme for further experiments and stopped the treatment after 3 (Figure S4) and 7 days (Figure 3A).

To validate senescence induction using these insults, we determined canonical senescence markers such as increased DNA damage response (DDR), cell cycle arrest, and SA-β-gal activity. Primary human lung fibroblasts exposed to bleomycin and H_2_O_2_ displayed increased nuclear expression of the DDR marker yH2Ax as well as CDKN1A/P21 after 7 days of treatment (Figure 3B). Moreover, bleomycin and H_2_O_2_ significantly induced SA-β-gal activity (Figure 3C) and the gene expression of *CDKN1A/P21*, whereas *CDKN2A/P16* was only significantly induced by H_2_O_2_ treatment (Figure 3D). Notably, TGF-β1 did not induce any of the evaluated senescence markers in phLF (Figure 3) but had a stronger effect inducing the expression of *PAI-1*, a well-known mediator of senescence (Figure 2F) as well as classical fibrotic markers (Figure S5). In conclusion, H_2_O_2_ and bleomycin induced a robust senescent phenotype in all phLF characterized by cell cycle inhibition, reduced proliferation, and increased SA-β-gal activity. On the other hand, TGF-β1 treatment led to increased fibrosis-related genes and the expression of well-known downstream mediators of the TGF-β1 signaling pathway like *PAI-1* but did not induce a senescent phenotype in phLF as judged by its effects on bona fide senescence markers.

### 3.4. Senescence Induction in phLF Is Not Impacted by Disease Background

**Figure 4:**
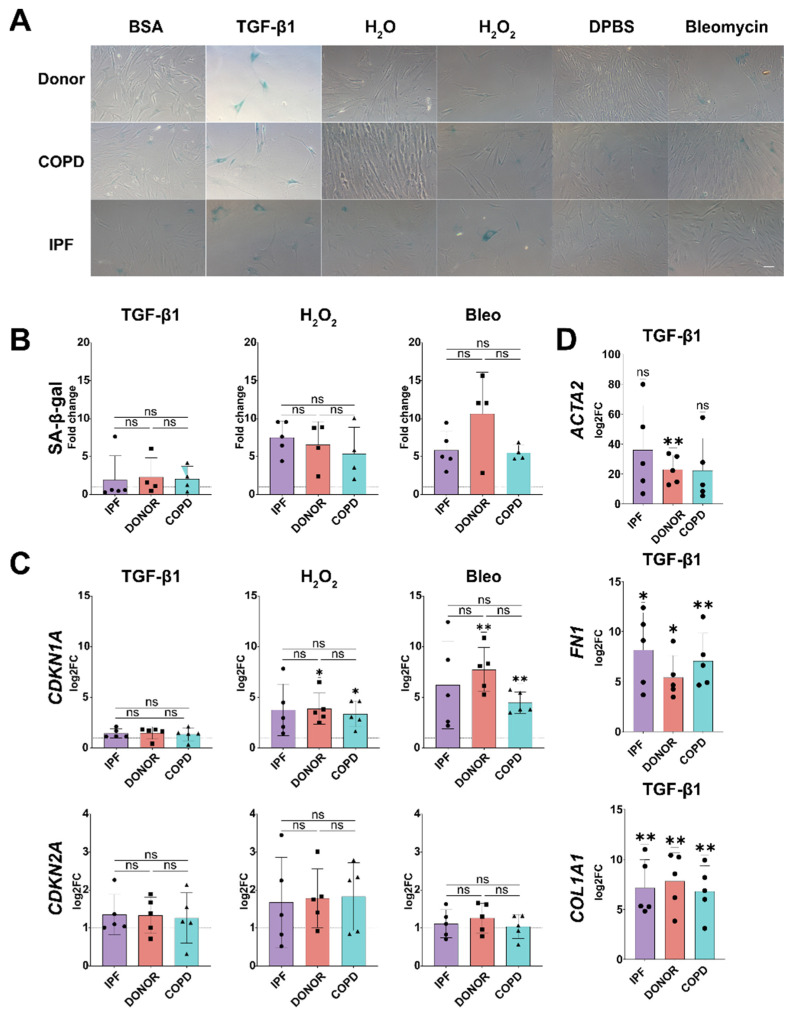
Primary human lung fibroblasts from different origins have a similar susceptibility to senescence and fibrotic stimuli. (**A**) Representative bright field images to determine senescence inducibility based on SA-β-galactosidase activity after 7 days of treatment with TGF-β1, H_2_O_2_, or bleomycin in phLF from Donor, COPD, and IPF patients. (**B**) Quantification of SA-β-galactosidase activity in phLF from Donor, COPD, and IPF patients after 7 days of treatment with TGF-β1, H_2_O_2_, or bleomycin, H_2_O_2_, bleomycin, or TGF-β1. (**C**) qRT-PCR to assess gene expression of senescence-related genes (*P21/CDKN1A, P16/CDKN2A*) in phLF from Donor, COPD, and IPF patients after 7 days of treatment with TGF-β1, H_2_O_2_, or bleomycin, H_2_O_2_, bleomycin, or TGF-β1. (**D**) qRT-PCR to assess gene expression of the fibrotic-related markers *ACTA2*, *FN1*, and *COL1A1* in phLF at 7 days after TGF-β1 treatment. Origin: * *p*-value < 0.05: Kruskal–Wallis test followed by Dunn´s multiple comparisons test. Log2FC to Ctrl: * *p*-value < 0.05, ** *p*-value < 0.01: One sample *t* test.

Given the transcriptomic differences in senescence markers observed in vivo in phLF derived from different disease backgrounds (Figure 1), we hypothesized that intrinsic factors in these phLF would determine their susceptibility to senescence induction. To test this, we exposed phLF derived from Control, IPF, and COPD lungs to the disease-relevant stimuli for 3 and 7 days (Figure 3). Here, we did not find any significant differences in SA-β-gal activity among non-disease/disease origins (Figure 3A,B). Similarly, we did not observe any significant difference in gene expression of cell cycle regulators: *CDKN1A/P21* or, *CDKN2A/P16* (Figure 3C). Classical TGF-β1 target genes also showed similar expression levels independent of disease background, suggesting that the cultured fibroblasts respond similarly to different stimuli (Figure 4D). In conclusion, we observed that phLF derived from different diseases are similarly susceptible to senescence induction.

### 3.5. Senescence Induction in phLF Is Stimuli-Specific

**Figure 5:**
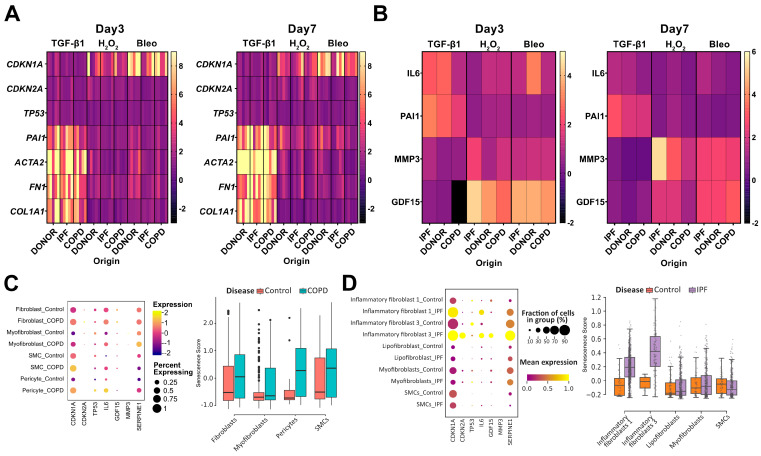
Different stimuli induce different senescence programs with in vivo relevance. (**A**) Heatmap of qRT-PCR to assess gene expression of senescence- (*P21/CDKN1A*, *P16/CDKN2A*, and *TP53*) and fibrosis-(*ACTA2*, *PA-1*, Fibronectin (*FN-1)*, and *COL1A1*) related genes after treatment with H_2_O_2_, bleomycin, or TGF-β1 for 3 and 7 days. Single rows represent biological replicates from Donor-, IPF-, and COPD-derived phLF (**B**) Heatmap of the SASP of Donor-, IPF-, and COPD-derived phLF as assessed by ELISA after treatment with H_2_O_2_, bleomycin, or TGF-β1 for 3 and 7 days. Single rows represent the average expression of at least 4 biological replicates. The concentration of each secreted protein (pg/mL) was normalized to total lysate protein content (mg/mL). (**C**) Dot plot of the mean expression of single SASP-related genes (**left**) and boxplot of senescence-score calculated with SASP-related genes (**right**) in the mesenchymal compartment of Control, and COPD lungs. Dot size represents the percentage of cells expressing each gene. (**D**) Dot plot of the mean expression of single SASP-related genes (**left**) and boxplot of senescence-score calculated with SASP-related genes (**right**) in the mesenchymal compartment of Control, and IPF lungs. Dot size represents the percentage of cells expressing each gene.

Next, we wanted to determine whether different stimuli induce specific senescence programs. For this, we compared the senescence signatures of phLF induced by TGF-β1, bleomycin, or H_2_O_2_ after stratification by disease origin. Here, we observed that both H_2_O_2_ and bleomycin-induced *CDKN1A/P21* expression, whereas *CDKN2A/P16* was mainly induced after H_2_O_2_ treatment (Figure 5A). Moreover, H_2_O_2_ and bleomycin induced the expression of *PAI-1*, *ACTA2*, and Fibronectin 1 (*FN-1*) after 7 days of culture (Figure 5A). Conversely, TGF-β1 did not increase the evaluated senescence-related genes but predominantly induced the expression of the pro-fibrotic markers: *PAI-1*, *ACTA2*, *FN-1*, and *COL1A1* (Figure 5A).

We then characterized the secretion of selected SASP factors over the culture time. Here, we found that H_2_O_2_ and bleomycin strongly induced GDF-15 secretion on day 3, whereas IL-6 was strongly induced only by TGF-β1 and bleomycin treatment (Figure 5B). PAI-1 secretion was only induced by TGF-β1 and sustained over culture time (Figure 5B). On the contrary, MMP3 secretion was mainly induced by H_2_O_2_ after 7 days (Figure 5B). In general, SASP profiles were similar between H_2_O_2_, and bleomycin as shown by an initial induction of IL-6, later induction of MMP-3 secretion, and sustained GDF-15 secretion (Figure 5B). Supporting a mainly pro-fibrotic effect, TGF-β1 only induced the secretion of PAI-1 and IL-6, well-known downstream factors of the TGF-β1 signaling pathway (Figure 5B). In conclusion, we observed specific senescence programs mostly depending on the senescence trigger. To validate, if these senescence programs are found in CLDs in patients, we analyzed the expression of these SASP factors in the single-cell RNA sequencing datasets from Control, COPD, and IPF patients as described in Figure 1. Here, we found the upregulation of *P21/CDKN1A*, *P16/CDKN2A*, *IL-6*, *GDF-15*, and *PAI-1*(*SERPINE-1*) to be increased in both COPD and IPF patients when compared to controls (Figure 5C,D). Interestingly, the upregulation of these SASP factors was found to be stronger in specific mesenchymal populations: fibroblasts, pericytes, and smooth-muscle cells (SMCs) for COPD and inflammatory fibroblasts 1 and 3 for IPF (Figure 5C,D). Highlighting, that the in vitro-induced senescence phenotypes are relevant to in vivo phenotypes found in CLDs.

### 3.6. Senescent Fibroblasts Disrupt Progenitor Potential of Distal Alveolar Epithelial Cells

**Figure 6:**
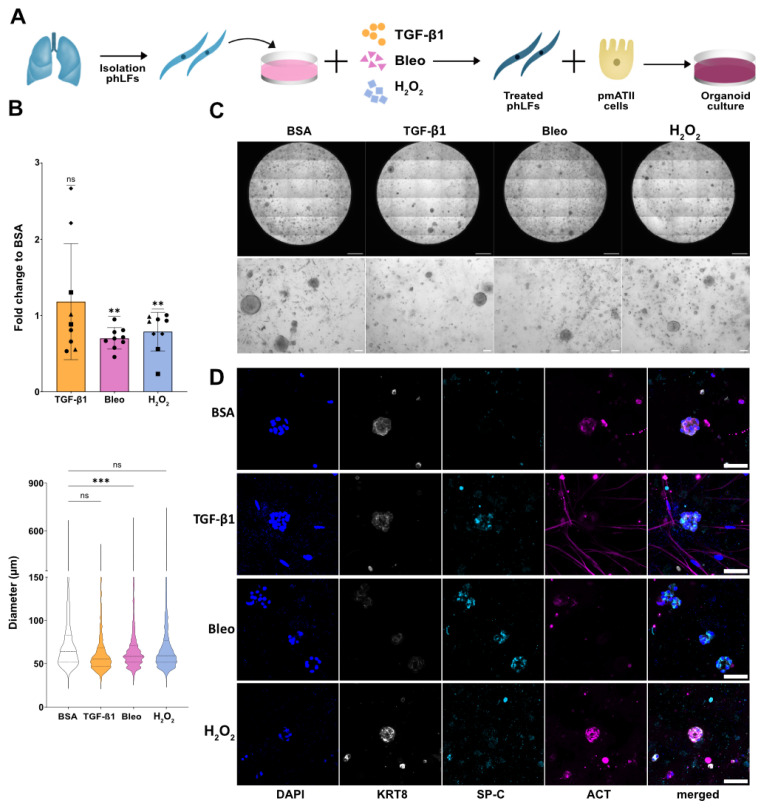
Senescent fibroblasts disrupt progenitor potential of lung epithelial cells. (**A**) Experimental design. Primary mouse lung epithelial cells were isolated and co-cultured with primary human fibroblasts (Pre-treated with H_2_O_2_, bleomycin, or TGF-β1 for 7 days) for 14 days in an organoid assay. (**B**) Fold change to BSA control for colony formation efficiency (CFE, top) and average spheroid size quantification (bottom). Single points represent 4 biological replicates (marked by shape) with 2 technical replicates for each. ** *p*-value < 0.01, based on a One-sample t-test (CFE) and *** *p*-value < 0.001 based on One way ANOVA test (size). (**C**) Representative bright-field images of whole wells and regions of interest. Scale bar 100 um. (**D**) Fluorescence images of single organoids (bottom) stained for surfactant protein C (SP-C), Acetylated Tubulin (ACT), and Keratin-8 (Krt8). Scale bar 100 µm.

In the alveolar niche, the epithelial and mesenchymal compartments closely interact. With age and in diseased lungs there is an impaired regenerative process in part mediated by alveolar epithelial progenitor cells. However, whether this is related to the presence of senescent lung fibroblasts is still understudied. To explore this, we co-cultured control or senescent phLF with distal alveolar epithelial progenitor cells in an organoid assay (Figure 6A) and determined both colony formation capacity as well as the cellular composition of the organoids formed. After 14 days, we observed that alveolar epithelial progenitor cells co-cultured with phLF pre-treated with bleomycin or H_2_O_2_ displayed a significant reduction in colony formation efficiency (CFE) in comparison with vehicle-treated controls (Figure 6B). Moreover, bleomycin-treated phLF significantly reduced the organoid size in comparison to control (Figure 6B). Finally, to characterize the cellular composition of the formed organoids, we stained them for surfactant protein C (SP-C), a marker for alveolar type 2 (AT2) cells, Keratin-8 (Krt8), a transdifferentiation marker from AT2 to AT1 cells, and acetylated Tubulin (ACT), a marker for airway epithelium. Here, we found the formation of alveolar (small and dark and surfactant protein C (SP-C) +), bronchiolar organoids (big with a lumen and acetylated Tubulin (ACT)+), and bronchoalveolar organoids (SP-C+/ACT+) in all conditions (Figure 6C,D). However, organoids derived from the co-culture with bleomycin-treated phLF showed lower Krt8 with a higher expression of SP-C in comparison with controls and other senescence inducers, suggesting impaired AT2 activation/differentiation (Figure 6D). In conclusion, the co-culture with senescent fibroblasts altered the stem cell capacity of alveolar epithelial progenitor cells in vitro.

## 4. Discussion

Aging is the main risk factor for CLDs such as COPD and IPF and previous studies have shown that senescent cells accumulate with age [35]. Although the mechanism is not fully understood, senescent cells can evade immune clearance, thereby accumulating and promoting organ dysfunction [36,37,38,39]. Indeed, elevated levels of CDKN1A/P21, CDKN2A/P16, and SA-β-galactosidase were found in fibroblasts from both IPF and COPD lungs and therefore, have been linked to the disease pathobiology [16,35,40,41,42]. However, whether the senescent phenotype is different depending on the disease background is not well understood. Therefore, in this study, we aimed to characterize the senescence of phLF from control, IPF, and COPD patients at baseline and after exposure to different senescence inducers. Finally, we used an organoid assay to study the crosstalk between epithelial and mesenchymal cells in vitro.

First, we characterized classical markers of senescence in situ and showed an increase in CTHRC1A+ P16+ cells. Similarly, mesenchymal cells in single-cell RNA sequencing from fresh single-cell suspensions from lung tissue showed increases in cellular senescence markers. However, phLF isolated from control, IPF, and COPD and cultured in cell culture had a very similar phenotype characterized by low expression of the different senescence-related markers: *CDKN1A/P21*, *CDKN2A/P16*, and *TP53* gene expression, SA-β-galactosidase activity, and secretion of SASP-related components. This suggests that by isolating and culturing the cells, we lose senescent cells due to cell competition or because cell phenotypes are significantly changed upon culturing on a plastic plate thereby reducing differences attributed to disease state as observed previously [43]. For example, senescence markers increased with prolonged culture as described for replication-induced senescence [44]. At the same time, the small number of patients included in this study represents a limitation. Previous studies showed that fibroblasts originating from COPD patients had an elevated senescence signature as judged by enhanced expression of CDKN1A/P21 and CDKN2A/P16, increased SA-β-galactosidase activity [45,46], reduced proliferation rates [42,46], and higher secreted levels of proteins associated with the SASP [15]. Moreover, COPD-derived fibroblasts inhibit canonical WNT-β-catenin signaling in alveolar epithelial cells by secreting WNT-5A, leading to stem cell exhaustion and impaired lung repair [47]. Similarly, phLF obtained from IPF lung tissue, showed decreased proliferation rates, increased expression of CDKN1A/P21, CDKN2A/P16, and TP53 as well as senescence-related morphological changes [16]. However, in the present study, we did not observe any major differences in senescence markers among disease origin, as reported previously [16,42,45,46]. This can be explained by different factors. For example, by different isolation protocols: in this study, phLF were isolated by enzymatic digestion contrary to the outgrowth from lung tissue pieces used in other studies [45]. Furthermore, intrinsic characteristics like the smoking history or passage number, as well as different culturing conditions and media supplementation can influence senescence readouts [48]. Finally, the composition of the isolated phLF can also vary depending on different anatomical localizations such as airway [46,49] versus whole lung [16,42] and current isolation methods do not discriminate among the different fibroblast subtypes recently described for the lung. In the past decades, it was believed that ACTA2+ positive myofibroblasts were the main contributor to ECM deposition in the IPF lung [19]. However, recent single-cell-based studies have revealed that IPF lungs have a higher heterogeneity in fibroblasts than control lungs and that these subpopulations coexist in the lung and might play distinct roles in the disease progression [17,19]. In this study, we identified inflammatory and CTHRC1+ fibroblasts as positive for senescence markers in situ in the lung from IPF and COPD patients. CTHRC1+ cells have recently been described as a marker for profibrotic fibroblasts mainly in IPF [32]. Interestingly, the susceptibility to typical fibrotic and senescence inducers such as bleomycin, has been shown to differ among these fibroblast subpopulations in the mouse lung [19]. Accordingly, the response to the stimuli used in this and other studies might be defined by the composition of the isolated and treated fibroblast population. The development of isolation protocols for primary lung fibroblasts based on the newly described markers would help to find specific disease-relevant cellular responses of these subpopulations that could be therapeutically targeted.

Cellular senescence can be induced by several stimuli such as increased oxidative stress, caused by exposure to cigarette smoke, or genomic DNA damage, induced by chemotherapeutic agents. Therefore, we used H_2_O_2_ and bleomycin to mimic these insults in vitro. Moreover, we also included TGF-β1, since it has been shown to induce a senescent phenotype in phLF [16,33]. In our study, only H_2_O_2_ and bleomycin induced cell cycle arrest as measured by *CDKN1A/CDKN2A* gene expression and other senescence-related markers such as reduced proliferation, increased SA-β-galactosidase activity, and increased secretion of GDF-15 and MMP-3 after 7 days. Notably, TGF-β1 only induced the expression of pro-fibrotic markers (*ACTA2*, *FN-1*, *COL1A1*, and *PAI-1*) as well as secretion of IL-6 and PAI-1 but did not induce a senescence phenotype defined by bona fide senescence markers. This could be explained by differences in the dosage, since in previous studies much higher doses were used to observe TGF-β1-induced senescence [33]. Previous studies showed that aged individuals have around 3–4 ng/mL circulating TGF-β1 in plasma [50]. Therefore, based on results using a more physiologically relevant dose (5ng/mL), we propose TGF-β1 as a pro-fibrotic rather than a senescence stimulus.

Next, we addressed whether the susceptibility to senescence was different among the different disease origins. Here, we found that IPF-derived phLF had a trend towards a reduced response to all stimuli in comparison to Donor- and COPD-derived phLF as previously described [51]. However, as observed at baseline, we did not find any significant difference in the senescence response among the different disease origins. This could be attributed to the chosen effective treatment regimens, which consistently induce a senescent phenotype overriding the cell origin. In conclusion, the senescence response of phLF is mainly defined by the trigger, in this case, DNA damage and oxidative stress, rather than by cellular predispositions. Interestingly, we observed similar gene expression and SASP profiles for bleomycin and H_2_O_2_ that differ from the one induced after TGF-β1. However, we also observed differences in the effect size for the tested markers. For example, the gene expression of *P16/CDKN2A* or the secretion of MMP-3 was more pronounced on H_2_O_2_-treated phLF. Therefore, a more comprehensive analysis of gene expression changes and secreted factors might be useful to better understand the differences between these two senescence inducers.

Senescent cells can modulate their microenvironment in a paracrine manner by their SASP or direct cellular interactions [35,52]. Therefore, we assessed the secretion of proteins related to inflammation and ECM deposition after induction of senescence. Here, we found that bleomycin and H_2_O_2_ induced the secretion of GDF-15, MMP-3, and PAI-1. Here, we could also find these SASP factors in single-cell sequencing datasets in the mesenchymal compartment of IPF and COPD patients, suggesting that our in vitro-induced senescence phenotypes are relevant to the in vivo situation. Indeed, in IPF lungs, MMP-3 is secreted by different cell types, including fibroblasts, and has been linked to lung epithelium dysfunction and poor regenerative capacity as well as fibroblast activation [53,54]. GDF-15 and PAI-1 are well-known SASP factors that also have been linked to inflammation and ECM remodeling in the diseased lung [55,56,57]. Interestingly, as previously described for paraquat-induced cellular senescence of lung fibroblasts [45], here we also observed that bleomycin and H_2_O_2_ decreased the expression of *COL1A1* in phLF. Moreover, bleomycin and H_2_O_2_ induced the gene expression of *PAI-1* and *FN-1*. This all together suggests that senescent fibroblasts can contribute to ECM remodeling as seen in CLDs.

Given the fact that senescent phLF can modulate their microenvironment by secreting pro-inflammatory and ECM-related proteins, we used an organoid assay to evaluate whether co-culture with them would alter the stem cell function of distal alveolar progenitor cells. Here, we found that both bleomycin and H_2_O_2_-induced senescence significantly reduced progenitor cell capacity as assessed by colony-forming efficiency. However, only bleomycin significantly altered the size of the formed organoids. These could be attributed to differences in the SASP and ECM-related gene expression between bleomycin- and H_2_O_2_-induced senescence programs in phLF. Further studies focusing on a comprehensive characterization of these factors could provide insights into the specific phLF-derived factors modulating the alveolar progenitor function in vitro. Moreover, although we did not observe any differences in the senescent phenotype depending on the origin, it would be of great interest to explore whether intrinsic factors associated with the disease background would also alter progenitor capacity in our organoid assay. Finally, senescent fibroblasts form only part of the niche that affects alveolar regenerative capacity, and the interactions with other niche factors including immune cells would be of great interest.

## 5. Conclusions

In conclusion, this study provides novel insights into the senescence phenotype of primary human lung fibroblasts exposed to disease-relevant insults. Moreover, in vitro organoid assays revealed that senescent phLF modulate the regenerative capacity of the lung progenitors. Further characterization of these phenotypes using state-of-the-art techniques such as single-cell sequencing could help elucidate the underlying mechanism that defines these senescence programs.

## Figures and Tables

**Table 1:** Patient demographics and clinical data. n/a: no information available.

Sex	Age	Smoker	Diagnosis
female	72	ex-smoker	Ctrl Donor
female	67	smoker	Ctrl Donor
male	84	ex-smoker	Ctrl Donor
female	57	smoker	Ctrl Donor
female	56	ex-smoker	IPF
male	72	n/a	IPF
female	54	never smoker	IPF
male	73	n/a	IPF
male	63	ex-smoker	IPF
male	67	ex-smoker	COPD
male	73	ex-smoker	COPD
female	69	ex-smoker	COPD
male	62	ex-smoker	COPD
female	n/a	n/a	COPD
male	64	smoker	COPD
male	58	smoker	COPD
female	60	smoker	COPD
female	61	smoker	COPD
unknown	n/a	n/a	Ctrl Donor
unknown	n/a	n/a	Ctrl Donor
unknown	n/a	n/a	Ctrl Donor
unknown	n/a	n/a	Ctrl Donor
male	58	ex-smoker	IPF
male	63	ex-smoker	IPF
male	n/a	never smoker	IPF
male	61	n/a	IPF
female	29	never smoker	Ctrl Donor
female	71	ex-smoker	Ctrl Donor
male	59	ex-smoker	Ctrl Donor
male	49	never smoker	Ctrl Donor
male	62	ex-smoker	COPD
male	58	ex-smoker	COPD
male	60	ex-smoker	COPD
male	65	ex-smoker	COPD
male	57	ex-smoker	IPF
female	40	never smoker	IPF
male	52	ex-smoker	IPF
male	62	ex-smoker	IPF
male	59	ex-smoker	IPF

**Table 2:** Primers for RT-qPCR.

Primer	Sequence (5′-3′)
ACTA2_fw	CGAGATCTCACTGACTACCTCATGA
ACTA2_rv	AGAGCTACATAACACAGTTTCTCCTTGA
FN-1_fw	CCGACCAGAAGTTTGGGTTCT
FN-1_rv	CAATGCGGTACATGACCCCT
COL1a1_fw	CAAGAGGAAGGCCAAGTCGAG
COL1a1_rv	TTGTCGCAGACGCAGATCC
PAI-1 fw	GACATCCTGGAACTGCCCTA
PAI-1 rv	GGTCATGTTGCCTTTCCAGT
CDKN2A_fw	ACCAGAGGCAGTAACCATGC
CDKN2A_rev	CCTGTAGGACCTTCGGTGAC
CDKN1A_fw	GTCAGTTCCTTGTGGAGCCG
CDKN1A_rev	TGGGTTCTGACGGACATCCC
TP53_fw	CGCTTCGAGATGTTCCGAGA
TP53_rv	CTTCAGGTGGCTGGAGTGAG
HPRT_fw	AAGGACCCCACGAAGTGTTG
HPRT_rv	GGCTTTGTATTTTGCTTTTCCA

**Table 3:** Antibodies used for immunofluorescence staining.

Target Protein	Host	Company	Ref. No
P21	rabbit	Abcam	ab109520
Phospho-histone H2A.X	mouse	Millipore	05–636
ACT	mouse	Abcam	ab24610
SP-C	rabbit	Abcam	ab3786
Krt8	rat	DSHB	TROMAI
Anti-rat-488	donkey	Invitrogen	A21208
Anti-rabbit-647	donkey	Invitrogen	A31573
Anti-mouse-568	donkey	Invitrogen	A10037
Anti-mouse-555	goat	Invitrogen	A21424
Anti-rabbit-488	goat	Invitrogen	A11008

## Data Availability

All data generated or analyzed during this study are included in this published article.

## Supplementary Materials

The following supporting information can be downloaded at: https://www.mdpi.com/article/10.3390/cells13131129/s1, Figure S1: Baseline senescence of primary human lunf fibroblasts after 3 days of culture; Figure S2: Establishment of treatment regimen for primary human fibroblasts to induce senescence; Figure S3: Cell proliferation rates of primary human fibroblasts at baseline and after treatment with with H_2_O_2_, Bleomycin, and TGF-ß1; Figure S4: Induction of senescence in primary human fibroblasts with disease-relevant stimuli; Figure S5: Induction of fibrotic markers in primary human fibroblasts; Figure S6: Viability and metabolic activity in primary human fibroblasts.
