# Supplementary material for: Stimuli-Specific Senescence of Primary Human Lung Fibroblasts Modulates Alveolar Stem Cell Function
Source: Cells. 2024 Jun 29;13(13):1129. doi: 10.3390/cells13131129 (PMC11240317; doi:10.3390/cells13131129)
Supplement: Supplementary file 1 [file cells-13-01129-s001.zip › cells-3036210-supplementary.pdf]

Supplementary Figures

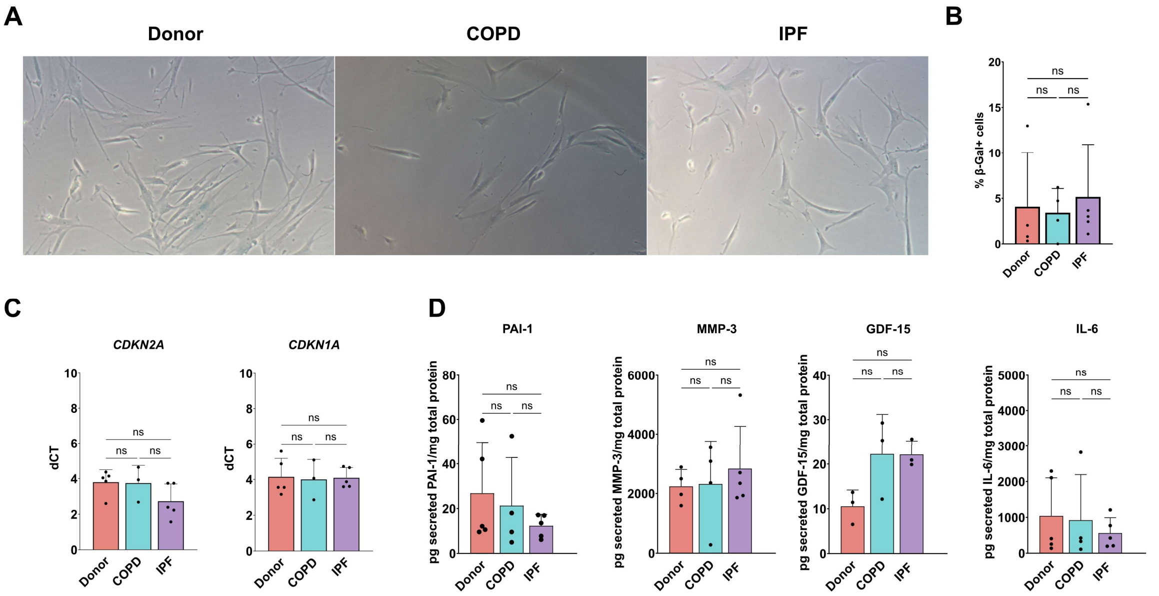

Figure S1. Baseline senescence of primary human lunf fibroblasts after 3 days of culture. A. Representative images SA-β-galactosidase staining of phLF from Donor, COPD, and IPF B. Quantification of SA-β-galactosidase staining of phLF from Donor, COPD, and IPF after 3 days of culture. Data points represent an average of 3 different regions of interest of at least 3 different biological replicates. C. qRT-PCR to assess gene expression of senescence-related genes (CDKN1A, CDKN2A) in phLF from Donor, COPD, and IPF patients after 3 days of culture. D.ELISA of phLF supernatants that were cultured for 3 days. Data points represent different biological replicates of the concentration of each secreted protein (pg/ml) normalized to total cell protein content (mg/ml). All p-values (<0.05) were calculated based on Kruskal-Wallis test.

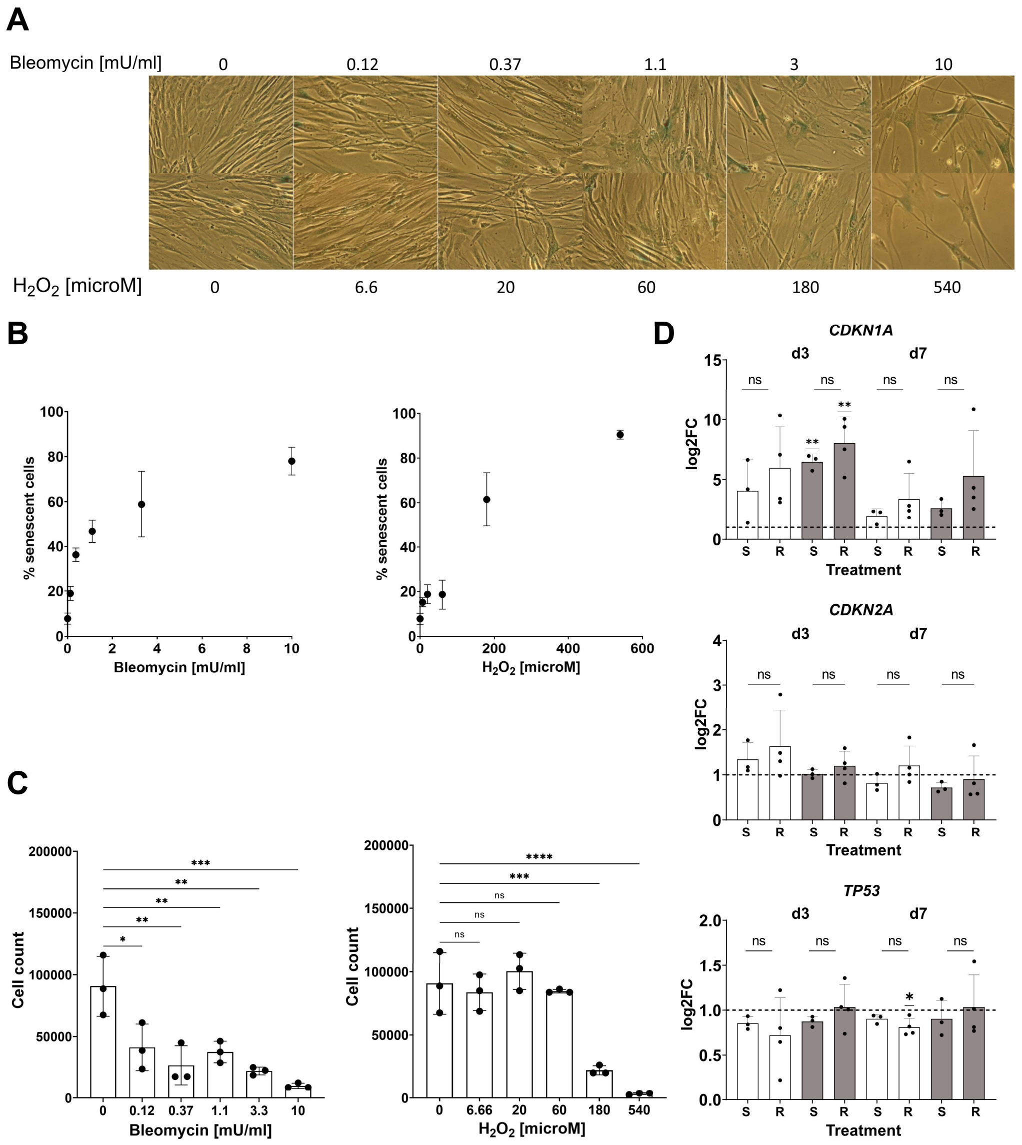

Figure S2. Establishment of treatment regimen for primary human fibroblasts to induce senescence. A. Representative images of SA-β-galactosidase staining after treatment of phLF with different concentrations of bleomycin and H_2O_2 to determine the best effective dose after 3 days of treatment. B. Titration of H_2O_2 and bleomycin concentration based on percentage of SA-β-galactosidase+ cells. C. Cell counts of phLF after treatment with different doses of bleomycin and H_2O_2. Single points represent replicates. *p-value<0.05 after one-way ANOVA test. D. qRT-PCR to assess gene expression of senescence-related markers after single (S) or repetitive (R) hit treatment with H_2O_2 (white) or bleomycin (gray) for 3 and 7 days. Single vs. repetitive hit: *p-value<0.05: Kruskal-Wallis test followed by Dunn´s multiple comparisons test. Log2FC to Ctrl: *p-value<0.05: One sample t test.

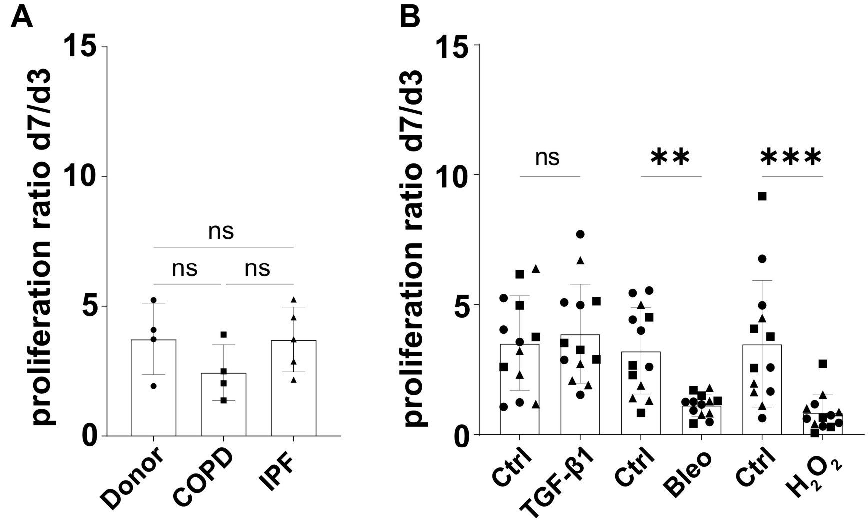

Figure S3. Cell proliferation rates of primary human fibroblasts at baseline and after treatment with with H_2O_2, Bleomycin, and TGF-ß1 . A. Proliferation rate (Cell count d7/d3) at baseline in phLFs isolated from IPF, COPD, and donor lungs. Data points represent biological replicates. *p-value<0.05: Kruskal-Wallis test followed by Dunn´s multiple comparisons test. B. Proliferation rate (Cell count d7/d3) after treatment with H_2O_2, bleomycin, and TGF-β 1. Data points represent biological replicates from donor (square), IPF (circle), and COPD (triangle). *p-value<0.05: Kruskal-Wallis test followed by Dunn´s multiple comparisons test.

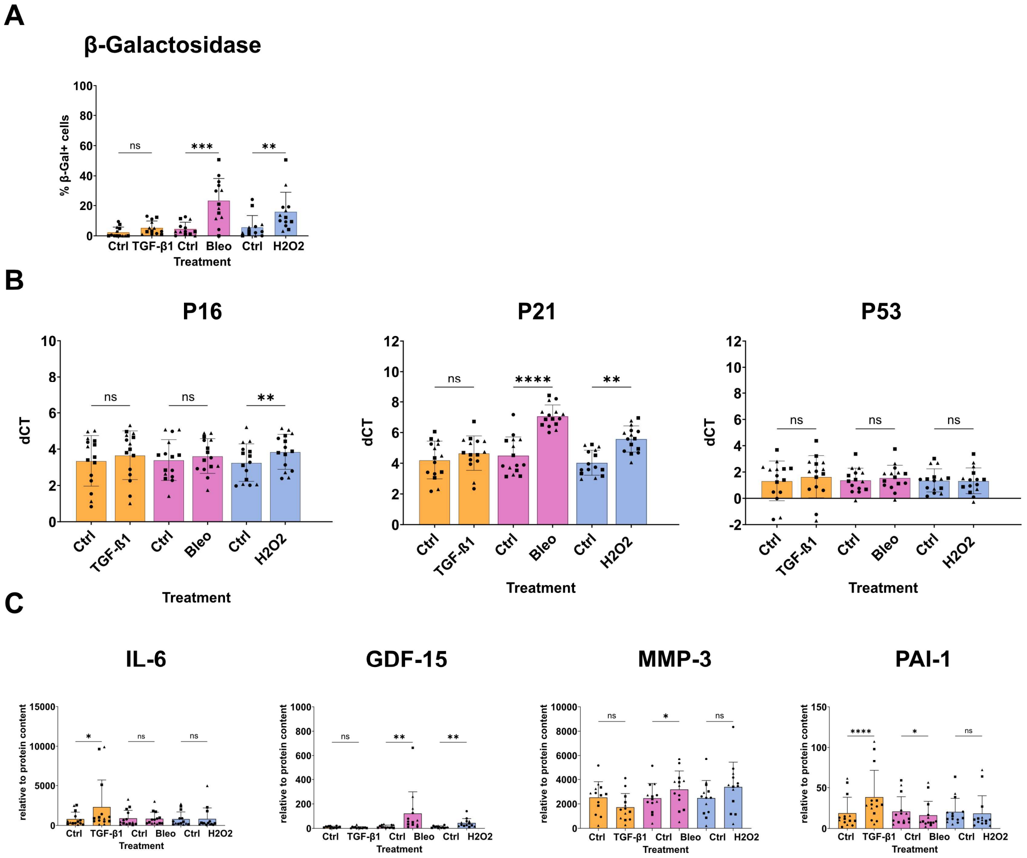

Figure S4. Induction of senescence in primary human fibroblasts with *disease-relevant* stimuli. A. Quantification of SA-β-galactosidase activity after 3 days of treatment. B. qRT-PCR to assess gene

expression of senescence-related markers after treatment with H_2O_2, bleomycin, and TGF-β1. Data points represent biological replicates from donor (square), IPF (circle), and COPD (triangle) *p-value<0.05: Friedman paired-test followed by Dunn´s multiple comparisons test. C. ELISA of supernatants of phLF treated with H_2O_2, bleomycin, and TGF-β 1 for 3 days. Data points represent different biological replicates from donor (square), IPF (circle), and COPD (triangle) of the concentration of each secreted protein (pg/ml) normalized to total lysate protein content (mg/ml). *p-value<0.05: Friedman paired-test followed by Dunn´s multiple comparisons test.

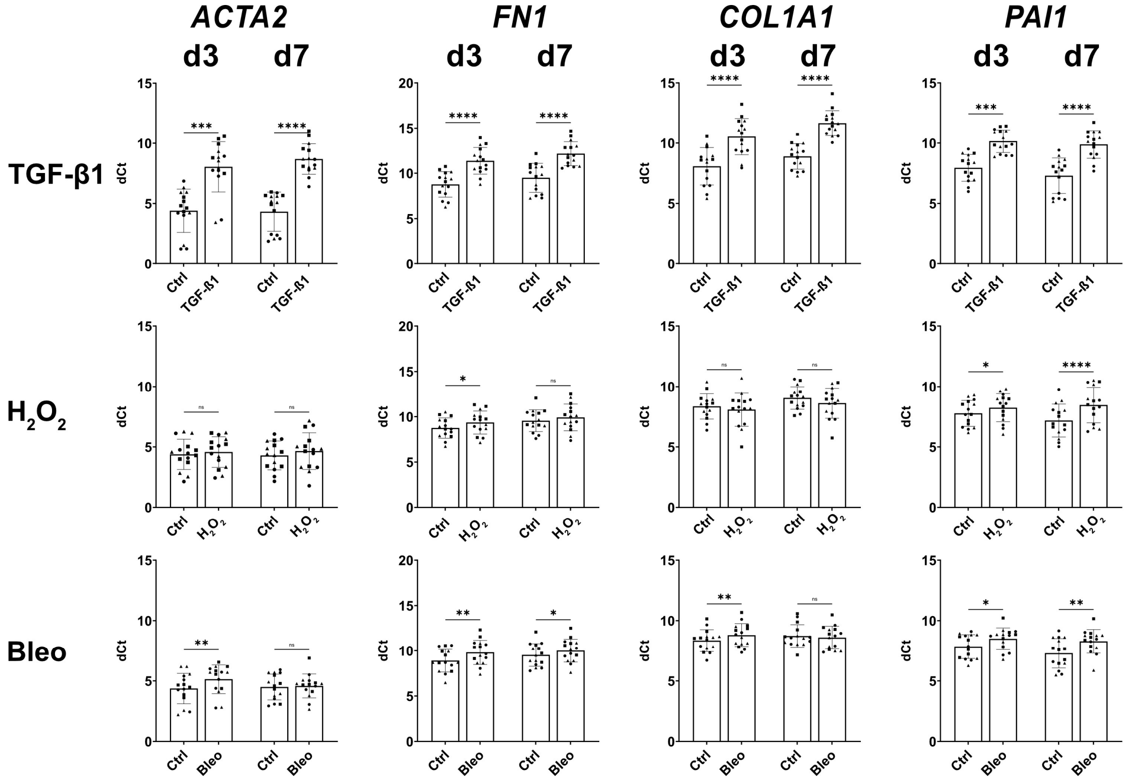

Figure S5. Induction of fibrotic markers in primary human fibroblasts. Expression of fibrosis-related markers after treatment with H2O2, Bleomycin, and TGF-ß1 for 3 and 7 days. Data points represent biological replicates from donor (square), IPF (circle), and COPD (triangle). *p-value<0.05 based on paired-Friedman T test.
